# Comprehensive identification and expression analysis of *B-Box* genes in cotton

**DOI:** 10.1186/s12864-021-07770-4

**Published:** 2021-06-12

**Authors:** Zhen Feng, Mengyu Li, Yi Li, Xu Yang, Hengling Wei, Xiaokang Fu, Liang Ma, Jianhua Lu, Hantao Wang, Shuxun Yu

**Affiliations:** 1 State Key Laboratory of Cotton Biology, Institute of Cotton Research of CAAS, Anyang, 455000 China; 2 Zhengzhou Research Base, State Key Laboratory of Cotton Biology, Zhengzhou University, Zhengzhou, China

**Keywords:** *G. hirsutum*, BBX, flower bud differentiation, phytohormone, stress response

## Abstract

**Background:**

B-BOX (BBX) proteins are zinc-finger transcription factors with one or two BBX domains and sometimes a CCT domain. These proteins play an essential role in regulating plant growth and development, as well as in resisting abiotic stress. So far, the *BBX* gene family has been widely studied in other crops. However, no one has systematically studied the *BBX* gene in cotton.

**Results:**

In the present study, 17, 18, 37 and 33 *BBX* genes were detected in *Gossypium arboreum*, *G. raimondii*, *G. hirsutum* and *G. barbadense*, respectively, via genome-wide identification. Phylogenetic analysis showed that all *BBX* genes were divided into 5 main categories. The protein motifs and exon/intron structures showed that each group of *BBX* genes was highly conserved. Collinearity analysis revealed that the amplification of *BBX* gene family in *Gossypium spp.* was mainly through segmental replication. Nonsynonymous (*Ka*)/ synonymous (*Ks*) substitution ratios indicated that the *BBX* gene family had undergone purification selection throughout the long-term natural selection process. Moreover, transcriptomic data showed that some *GhBBX* genes were highly expressed in floral organs. The qRT-PCR results showed that there were significant differences in *GhBBX* genes in leaves and shoot apexes between early-maturing materials and late-maturing materials at most periods. Yeast two-hybrid results showed that *GhBBX5*/*GhBBX23* and *GhBBX8*/*GhBBX26* might interact with *GhFT*. Transcriptome data analysis and qRT-PCR verification showed that different *GhBBX* genes had different biological functions in abiotic stress and phytohormone response.

**Conclusions:**

Our comprehensive analysis of *BBX* in *G. hirsutum* provided a basis for further study on the molecular role of *GhBBXs* in regulating flowering and cotton resistance to abiotic stress.

**Supplementary Information:**

The online version contains supplementary material available at 10.1186/s12864-021-07770-4.

## Background

Zinc-finger transcription factors are a kind of vital proteins, which play essential roles in plant growth and development, as well as in response to environmental stimuli [1, 2]. B-BOX(BBX) protein is one of zinc-finger transcription factors, which have attracted increased amounts of attention in recent years because of its various functions. BBX proteins are characterized by one or two conserved BBX domains at their N-terminus and sometimes a CCT domain at their C-terminus. BBX domains play an important role in transcriptional regulation and protein-protein interactions [3, 4]. The CCT domain is involved in transcriptional regulation and nuclear transport [5–7]. In *Arabidopsis*, 32 BBX proteins have been identified. According to the existence of BBX domain and CCT domain, these members can be divided into five subgroups [3]. A growing body of evidence also shows that BBX proteins play a crucial role in flowering [8, 9], abiotic stress responses [10] and hormonal signaling networks[4].

*CO*/*AtBBX1* was the first BBX gene studied in *Arabidopsis*; this gene controlled flowering time by regulating the expression of downstream *Flowering Locus T* (*FT*) gene [11–13]. Flowering is significantly delayed in *CO* mutant plants, while overexpression of *CO* could make plants flower early [14, 15]. Other *BBX* genes subsequently discovered, such as *BBX4*, *BBX6*, *BBX7*, and *BBX32*, could also regulate flowering time [16–19]. The mutant plants of *BBX4* flower earlier than wild plants, indicating that *BBX4* can delay the flowering of plants [16]. The mutant plants of *BBX7* could also flower earlier, and the plants with overexpression of *BBX7* showing the phenotype of delayed flowering [17]. The overexpression of *BBX6* could make the plant flower earlier [19]. Overexpression of *BBX32* showed the opposite phenotype, which could delay the flowering of the plant [18].

BBX proteins also participate in abiotic stress responses and hormone signaling networks. For example, compared with the wild type, the overexpression of *BBX24* in *Arabidopsis* had higher salt tolerance, and the root length of *BBX24* transgenic plants increased significantly under high-salinity conditions [20]. In Chrysanthemum, overexpression *CmBBX24* not only prolonged flowering time, but also enhanced cold and drought resistance [13]. *BBX* genes also play a role in phytohormone signal transduction. *AtBBX18* is a positive regulator of the gibberellin (GA) signaling pathway. Molecular and phenotypic studies have shown that *BBX18* promotes hypocotyl growth by increasing bioactive gibberellin levels [21]. While *BBX20* is a negative regulator of brassinolide signal pathway. It promotes hypocotyl growth by directly binding *BZR1* and inhibiting its expression [22].

Cotton is an important cash crop species. Although members of the *BBX* family have been identified in *Arabidopsis*, tomato, pear, and apple [3, 23–25], no comprehensive study of *BBX* genes in cotton has been reported so far. With the release of the cotton genome[26], we can more systematically study the hypothetical functions of *BBX* genes in cotton. In the present study, we made a comprehensive analysis of the physical and chemical properties, chromosome distribution, collinearity, gene structures, cis-acting elements and expression patterns of the *BBX* gene family in *G. arboreum*, *G. barbadense*, *G. hirsutum* and *G. raimondii*. This research provided basic data for further study on the function of *BBX* genes in cotton.

## Results

### Identification, chromosomal distribution and subcellular localization of *BBX* gene family

**Fig. 1:**
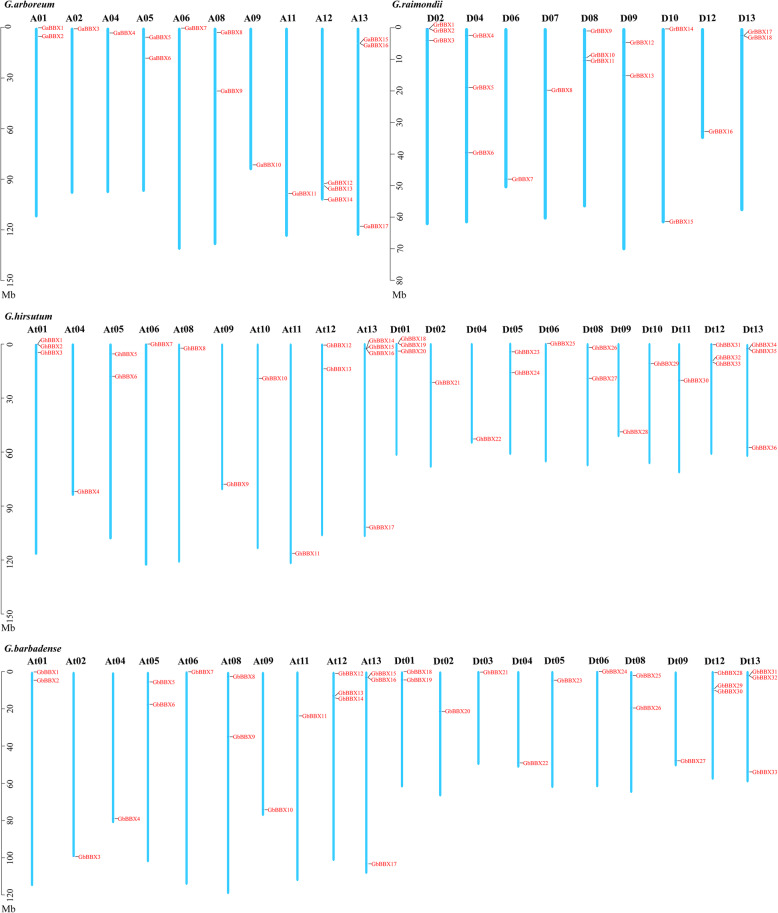
Chromosomal distribution of *BBXs* in *G. arboreum*, *G. raimondii*, *G. hirsutum* and *G. barbadense*. The chromosome numbers are presented above each vertical bar. The scale is in base pairs (Mb)

To identify the *BBX* genes in the *Gossypium spp*. genome and obtain their sequences, a global search of the *Gossypium spp*. genomes were carried out by using HMM profiling of the BBX domain (PF00643). After ensuring that the identified members contained conserved domains and deleted the repeated sequences, in total, of 17, 18, 37 and 33 putative BBX sequences were identified in *G. arboreum*, *G. raimondii*, *G. hirsutum* and *G. barbadense*, respectively, via genome-wide identification analysis. In *G. hirsutum*, 1 *BBX* was located on scaffold fragments. The *BBXs* were named according to their location on the chromosomes (Fig. 1), and the *BBXs* located on the scaffold fragments in *G. hirsutum* is finally named. Table S2 contained detailed location information. The lengths of putative GaBBX protein sequences ranged from 163 aa (GaBBX3) to 374 aa (GaBBX13); GrBBXs, 197 aa (GrBBX16) to 374 aa (GrBBX10); GhBBXs, 166 aa (GhBBX29) to 374 aa (GhBBX32) and GbBBXs, 136 aa (GbBBX3) to 505 aa (GbBBX27). The predicted MW and pI of each BBX were shown in Table S2. The results of subcellular localization showed that all of the *BBXs* were located in the nucleus, indicating that the nucleus was the main region of biological functions of *BBXs*.

Based on the genomic location information of 105 *BBX* genes, we visualized the chromosome distribution of *GaBBXs*, *GrBBXs*, *GhBBXs* and *GbBBXs* (Fig. 1). In *G. arboreum*, 17 *GaBBXs* were unevenly distributed on 10 chromosomes. A12 and A13 contained 3 *GaBBXs*, whereas the other 8 chromosomes, A01, A02, A04, A05, A06, A08, A09 and A11, contained 1 or 2 *GaBBXs*. In *G. raimondii*, 18 *GrBBXs* were located on 9 chromosomes. D02 and D08 contained the most *GrBBXs* (3), while the other 6 chromosomes contained only 1 or 2 *GrBBXs*. In *G. hirsutum*, 37 *GhBBXs* were unevenly mapped to 21 chromosomes, while, *GhBBX37* was located on unassembled scaffolds. At13 contained 4 *GhBBXs*. At01, Dt01, Dt12 and Dt13 contained 3 *GhBBXs*, and the other 16 chromosomes contained only 1 or 2 *GhBBXs*. In *G. barbadense*, 33 *GrBBXs* were unevenly mapped to 20 chromosomes. At12, At13, Dt12 and Dt13 contained 3 *GrBBXs*. The other 16 chromosomes contained only 1 or 2 *GrBBXs*.

### Phylogenetic analysis of the *BBX* gene family

**Fig. 2:**
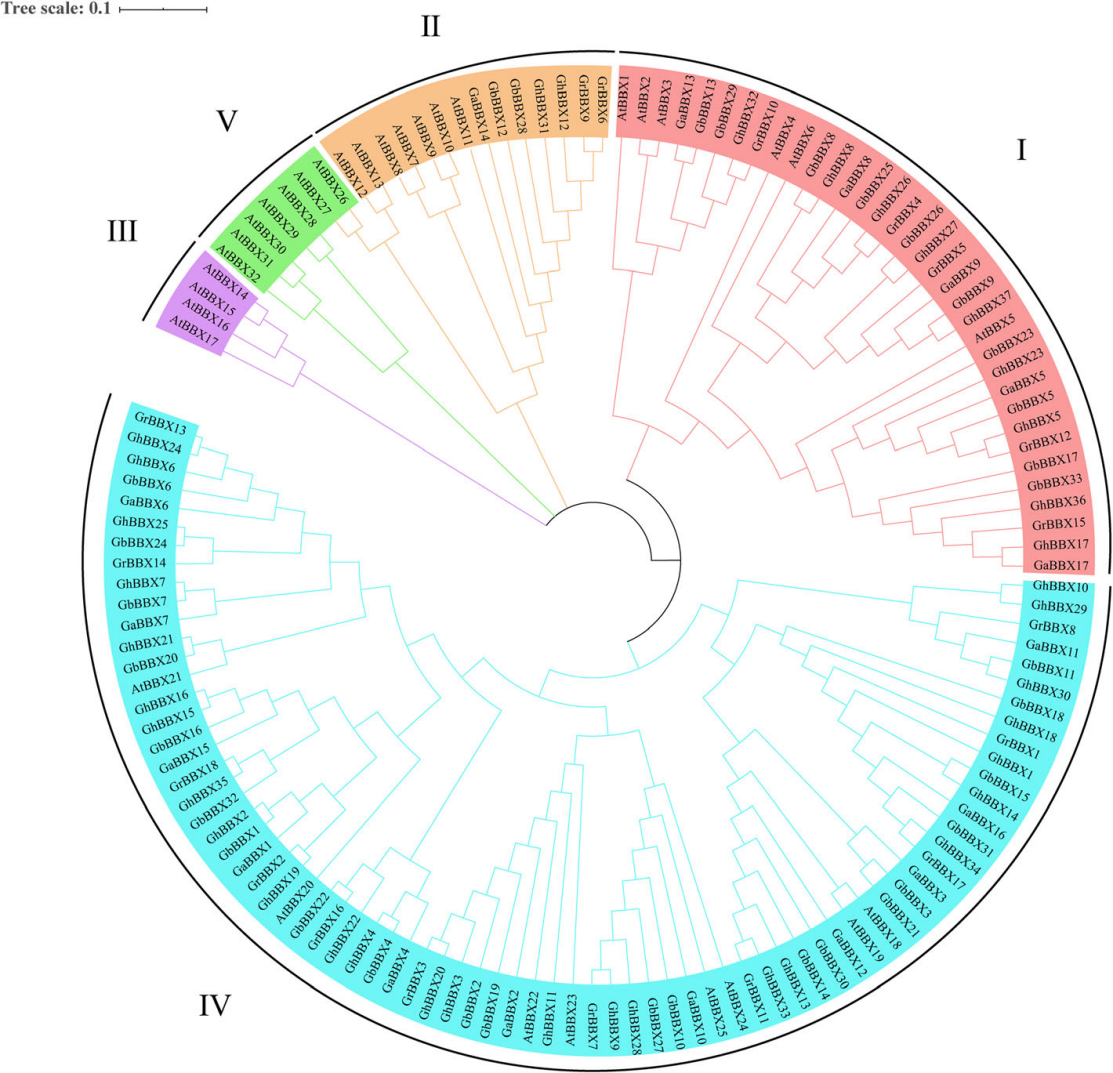
Phylogenetic tree of BBX proteins. The sequences of 105 BBX proteins of *G. raimondii*, *G. arboreum*, *G. hirsutum*, *G. barbadense* and *A. thaliana* (shown here) were aligned with ClustalX version 2.0, and a phylogenetic tree was generated by Mega 7.0 software using the NJ method with 1000 bootstrap replicates. Different colors present the five subfamilies of BBXs

To investigate the phylogenetic relationships of *BBXs*, 137 BBX protein sequences (*G. arboreum* (17), *G. raimondii* (18), *G. hirsutum* (37), *G. barbadense* (33) and *A. thaliana* (32)) were used to construct a phylogenetic tree based on the NJ method. Members of the *BBX* family were classified into 5 major groups, I-V (Fig. 2), and each subgroup was named according to the taxonomic results of previous studies in *Arabidopsis* [4]. It was worth noting that although *AtBBX26* and *AtBBX27* belonged to group V of *Arabidopsis* according to their structural classification, they were phylogenetically closer to *AtBBX12* and *AtBBX13*, which were in group II. As shown in Fig. 2, group II was the smallest subgroup, containing 7 *BBXs*. By contrast, group IV had the most massive numbers of *BBX* genes, including 69 *BBXs*. There were 29 *BBXs* in group I. No cotton species were divided into group III or group V. In *G. hirsutum*, *BBX* gene had 2, 9 and 26 members in group II, I and IV, respectively.

### Replication events of *BBX* gene family

**Fig. 3:**
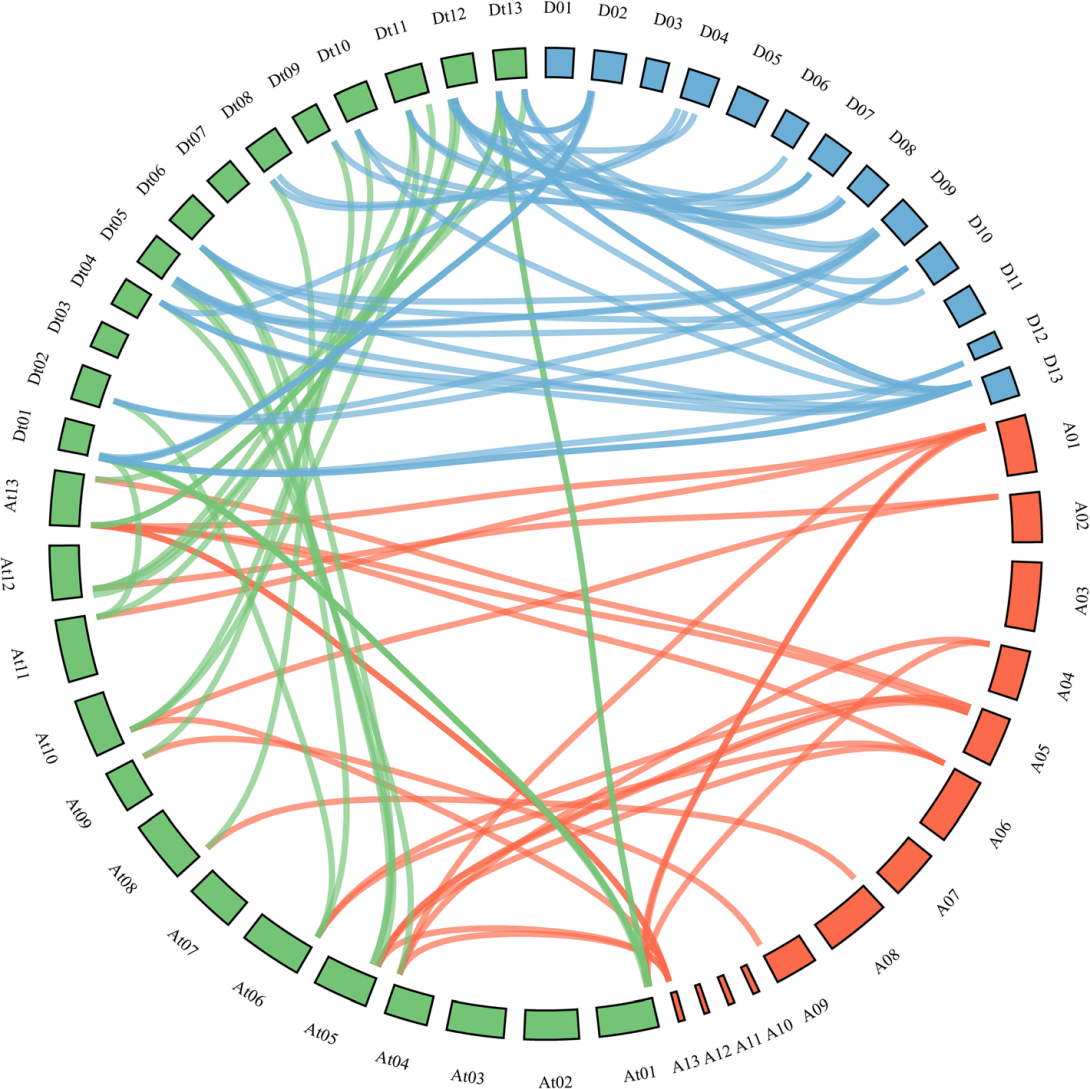
Genome-wide synteny results of *BBX* genes from *G. arboreum*, *G. raimondii*, and *G. hirsutum*. The red lines present linked gene pairs between *G. arboreum* and *G. hirsutum*. The blue lines present linked gene pairs between *G. raimondii* and *G. hirsutum*, and the green lines present linked gene pairs between *G. hirsutum* (A and D subgenomes)

From the perspective of the cotton evolution, tetraploid cotton is the result of genome doubling of two diploid cotton hybrids. In terms of the number of genes, the sum of *BBX* genes in *G. arboreum* (17) and *G. raimondii* (19) was about equal to the number of those in *G. hirsutum* (37) or *G. barbadense* (33). The results further confirmed this view. To explore the replication events of the *BBX* gene family, MCScanX was used to analyze the collinearity between the A_t_ and D_t_ subgenomes of *G. hirsutum* and their corresponding ancestral A and D diploid genomes (Fig. 3). The data showed that most homologous gene pairs of the *BBX* gene family were amplified by segmental replication, which meant segmental replication played a key role in the evolution of the *BBX* gene family. However, the genomic evolution of allotetraploid cotton is extremely complex. In the process of evolution, the genome has experienced not only segmental duplication events but also many tandem duplication events. The duplicate types of *BBXs* in *G. hirsutum* were shown in detail in Table S3. In *G. arboretum* and *G. raimondii*, 1 and 2 tandem duplication events (*GaBBX15*/*GaBBX16* as well as *GrBBX1/GrBBX2* and *GrBBX17/GrBBX18*) were identified, respectively. In *G. hirsutum*, 4 tandem duplications (*GhBBX1/GhBBX2*, *GhBBX15/GhBBX16*, *GhBBX18/GhBBX19*, *GhBBX34/GhBBX35*) were discovered.

*Ka*/*Ks* ratios were calculated to evaluate the selection pressure of these homologous gene pairs. Among the 95 homologous gene pairs, 90 homologous gene pairs had *Ka*/*Ks* values < 1, which indicated that most of the homologous gene pairs had undergone purifying selection in the process of evolution, and these genes pairs might play a similar function. Only a few homologous gene pairs had experienced positive selection, which might lead to new biological functions of these genes.

### Analysis of gene structure and conservative motif

**Fig. 4:**
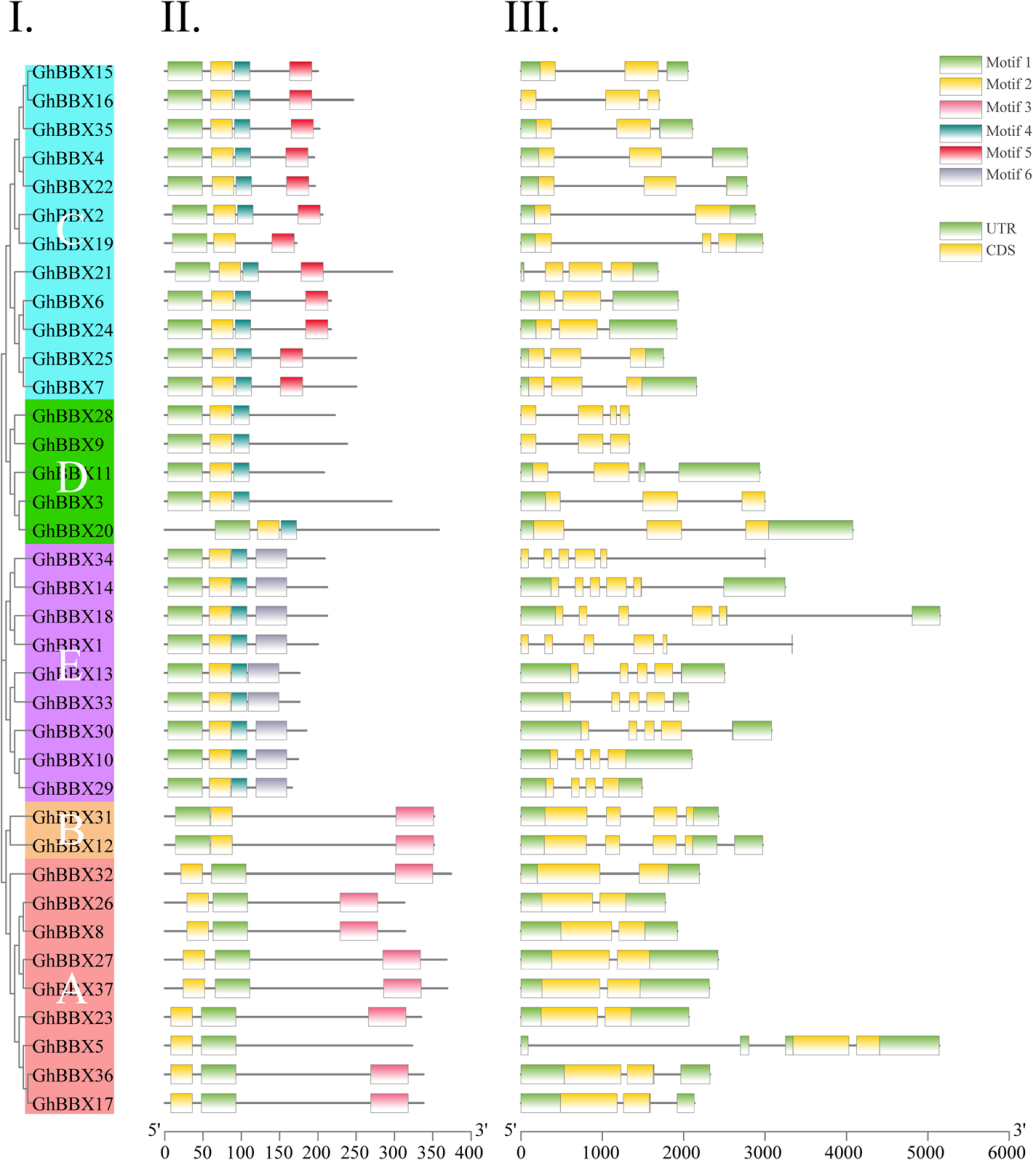
Gene structure and conserved protein motifs of *GhBBXs*. (**I**) NJ phylogenetic tree analysis of *G. hirsutum*. A-E represent the five subgroups. (**II**) Shown is the distribution of the predicted motifs in the *GhBBX* genes. (**III**) Shown are the number, length, and position of exons and introns within *GhBBX* genes. The boxes present exons, and the black lines present introns

The results of the phylogenetic analysis showed that 37 *GhBBXs* could be divided into 5 groups (A-E), which contained 9, 2, 12, 5 and 9 members, respectively (Fig. 4I). To better understand the structural characteristics of *GhBBXs*, the exon/intron structure was analyzed by GSDS (Fig. 4III). The *GhBBX* genes contained 3 to 7 exons, but most of them contained less than 5 exons. Moreover, the conserved motif was further analyzed by MEME program. The *GhBBXs* in the same group showed similar motif composition, which further validated the classification results (Fig. 4II). Except for group A, the order of motif 1 and motif 2 in *GhBBX* of other groups was the same. Motif 3 existed only in group A and group B, but motif 4 existed in all groups except group A and group B. Motif 5 existed only in group C, while motif 6 only existed in group E. Figure 4 showed that the distribution of conserved motif and exon/intron structure were different among different groups, but they were highly conservative on the same branches. The results showed apparent conservation, which laying a foundation for functional conservatism and providing guidance for follow-up functional research.

### Analysis of cis-acting elements in *GhBBX* promoter regions

To better understand the regulation of *GhBBXs* gene transcription and expression, the promoter region of *GhBBX* (genomic DNA sequence 2 kb upstream of the transcription start site) were used to search the PlantCARE database. A variety of cis-elements were found in the *GhBBX* promoter region. Among the cis-acting elements, the cis-acting elements related to phytohormone and stress response were the focus of our attention. We found abscisic acid (ABA) response element, gibberellin (GA) response element, auxin (IAA) response element, salicylic acid (SA) response element and methyl jasmonate (MeJA) response element in 21, 19, 11, 17 and 17 *GhBBX* promoters, respectively. In some *GhBBX* promoters, there were cis-acting element related to multiple phytohormone, while in other *GhBBX* promoters, there were only cis-acting element related to a single phytohormone response. In terms of stress-related response elements, these cis-acting elements were mainly related to low temperature, drought, anaerobic and other defenses. In the midst of these elements, the anaerobic cis-acting element was the most frequent stress response element, which appeared in the promoters of 32 *GhBBX* genes, followed by the cis-acting element in response to low temperature. It existed in the promoters of 20 *GhBBX* genes. Thus, it could be seen that *GhBBX* might respond to stress response and abiotic stress of cotton. In addition, a large number of light response elements were found in the promoter region of *GhBBXs*, including Box-4, G-box, GT1-motif, TCT-motif and MRE.

### Expression patterns of *GhBBXs* in different tissues

**Fig. 5:**
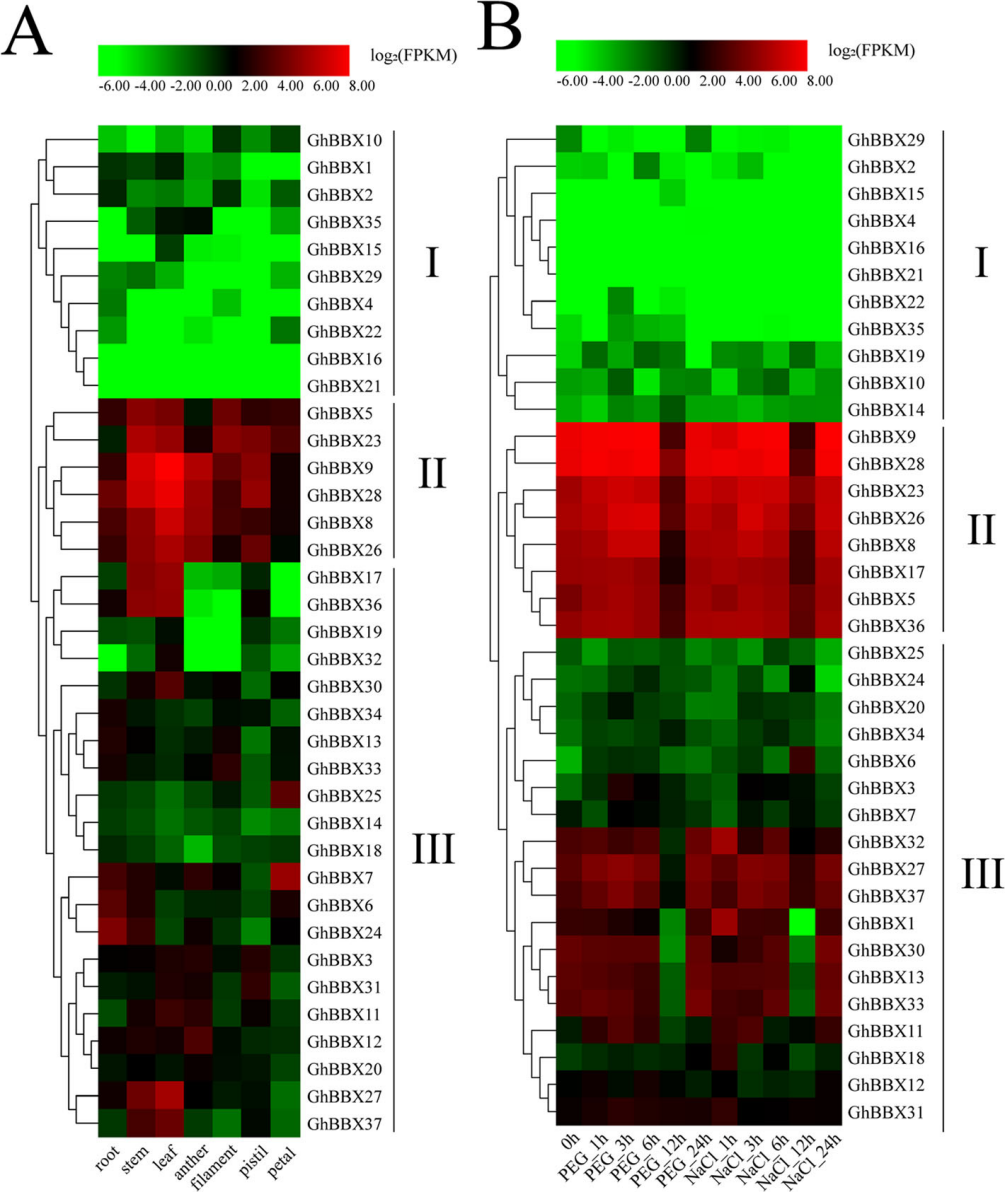
Expression profiles of *GhBBXs* in different tissues (**A**) and response to different stresses (**B**). The tissues or treatments are shown at the bottom, the genes are shown on the right, and the phylogenetic relationships are shown on the left

In order to study the expression pattern of *GhBBXs* in different tissues, we analyzed the transcriptomic data of root, stem, leaf, anther, filament, pistil and petal in TM-1. The results showed that different members of the cotton BBX family showed different expression patterns. According to the expression characteristics and based on hierarchical clustering analysis, 37 *GhBBXs* were divided into 3 categories (I-III) (Fig. 5). 6 *GhBBXs* (*GhBBX5*, *8*, *9*, *23 26*, and *28*) belonging to group II were highly expressed in nearly all tissues. 10 *GhBBXs* (*GhBBX2*, *4*, *10*, *16*, *19*, *21*, *22*, *29*, *32* and *35*) belonging to group I were poorly expressed in all tissues. The remaining members (*GhBBX1*, *3*, *6*, *7*, *11*, *12*, *13*, *14*, *15*, *17*, *18*, *20*, *24*, *25*, *27*, *30*, *31*, *33*, *34*, *36* and *37*) belonging to group III exhibited slightly higher expression in vegetative organs, while others showed slightly higher expression in floral organs. These differences in expression patterns might be related to the various functions of *GhBBXs*.

### Expression characterization of *GhBBXs* in cotton flower bud differentiation

**Fig. 6:**
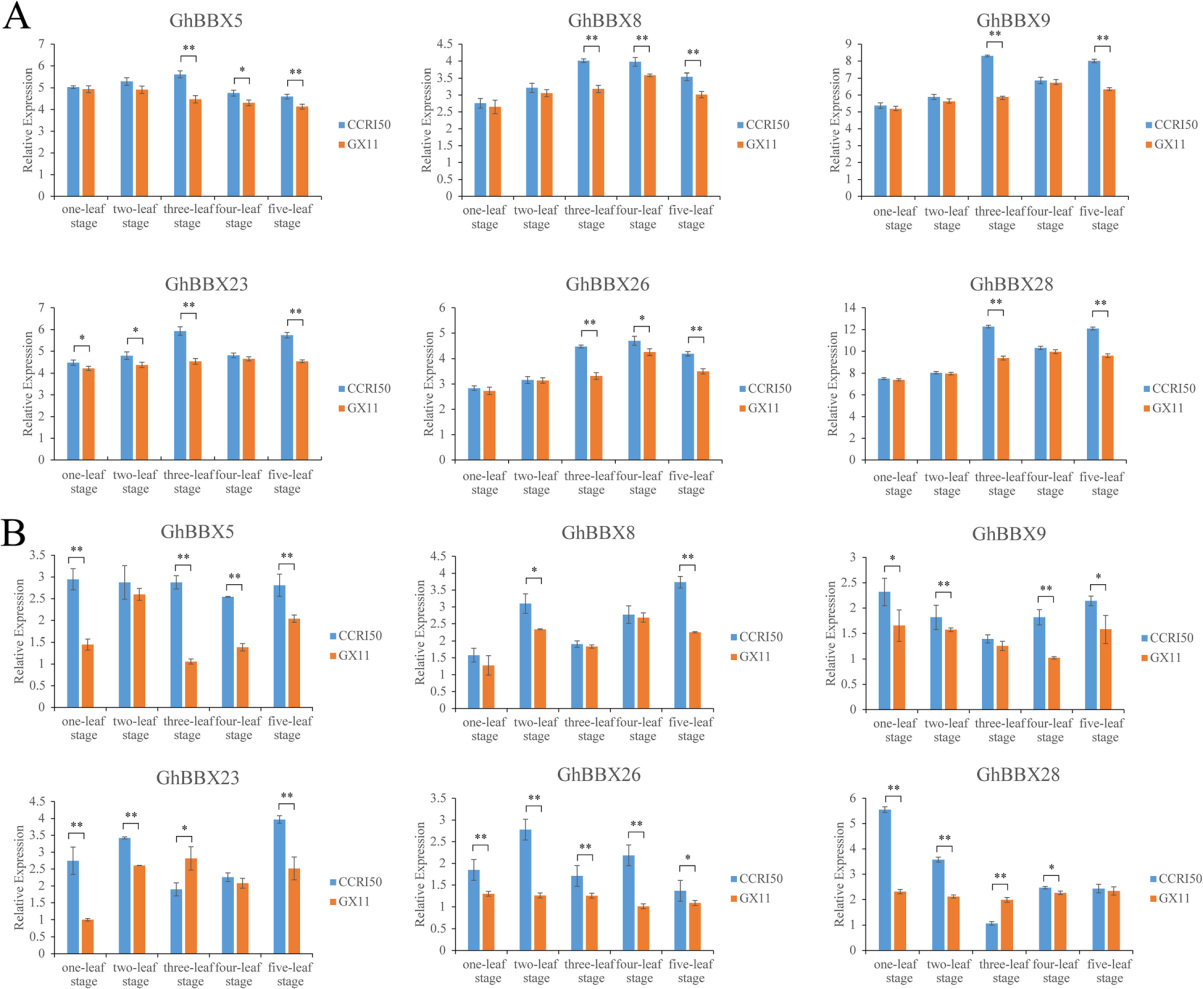
Gene expression levels from the one-leaf stage to five-leaf stage of CCRI50 and GX11. (**A**: Leaf, **B**: Shoot apex). The transcript levels of the 6 genes were analyzed by qRT-PCR. *GhActin* was used as an internal control. The gene expression levels are expressed as relative values, and the error bars present the means ± standard errors (SEs) of the three biological replicates. Significant differences are presented by “*” (*p* < 0.05). Extremely significant differences are presented by “**” (*p* < 0.01)

Flower bud differentiation is an important sign that a plant is undergoing a transition from vegetative growth to reproductive growth [27]. To explore whether *GhBBX* gene, which was highly expressed in flower organs, was involved in the process of flower bud differentiation, we analyzed the relative expression of these genes in the leaf and shoot apex of the early-maturing cotton cultivar CCRI50 and late-maturing cotton cultivar GX11 from one-leaf stage to five-leaf stage. The graphical representation of the expression profiles of 6 genes in the leaf and shoot apex at 5 different times was shown in Fig. 6. In the leaf, the expression levels of these six genes in the three-leaf stage and five-leaf stage of early-maturing materials were higher than those of late-maturing materials, and showed a significant difference. There was a difference in the expression of *GhBBX5*, *GhBBX8* and *GhBBX26* between early-maturing materials and late-maturing materials at four-leaf stage, but in one-leaf stage and two-leaf stage, only the expression of *GhBBX23* was different between early-maturing materials and late-maturing materials, there was no significant difference in the expression of the other five genes. In the shoot apex, the expression levels of *GhBBX5*, *GhBBX8*, *GhBBX9*, and *GhBBX26* in the early-maturing material were higher than those in the late-maturing material, and significant or extremely significant differences were detected at 4, 1, 3, and 5 periods, respectively. The expression levels of the other 2 genes, *GhBBX23* and *GhBBX28*, in the early-maturing material were higher than those in the late-maturing materials at the most stages, but at three-leaf stage, the expression levels in the early-maturing material were lower than those in the late-maturing material.

### *GhBBXs* interact with key genes in cotton flowering pathway

**Fig. 7:**
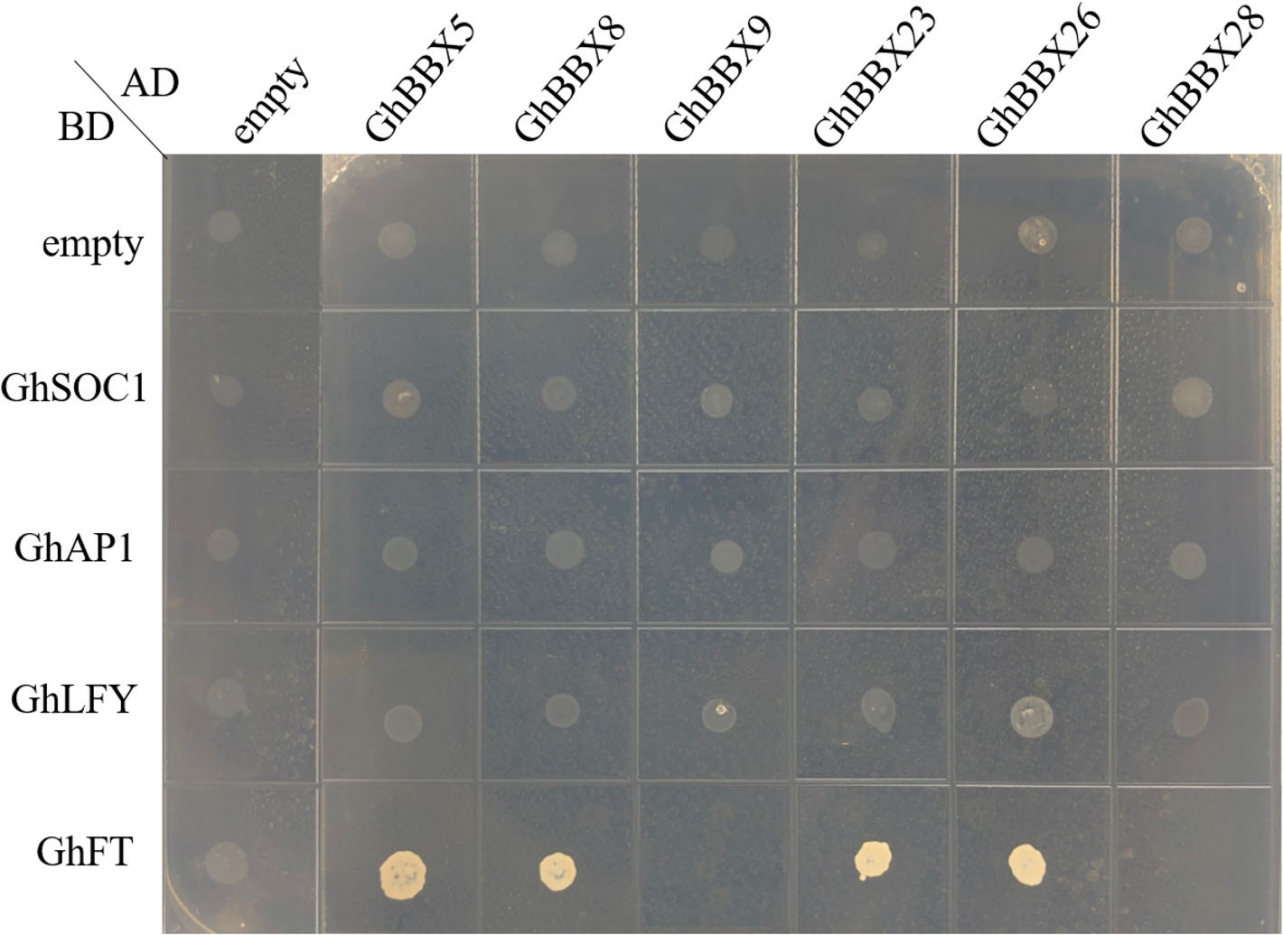
Yeast two-hybrid assay of interaction between six *GhBBXs* and *GhFT*, *GhSOC1*, *GhLFY* and *GhAP1*. Yeast cells were co-transformed with recombinant pGADT7 and pGBKT7 vectors and grew on the SD-Trp/−Leu/−His/−Ade/ medium

The previous studies showed that *GhFT*, *GhSOC1*, *GhLFY* and *GhAP1* might be key factors in cotton flowering pathway [28–31]. In order to explore the interaction of *GhBBXs* with key regulatory factors in flowering pathway, we selected *GhBBX5*, *GhBBX8*, *GhBBX9*, *GhBBX23*, *GhBBX26* and *GhBBX28* to detect their interaction with *GhFT*, *GhSOC1*, *GhLFY* and *GhAP1* by yeast two-hybrid assay. The results showed that *GhBBX5*, *GhBBX8*, *GhBBX23* and *GhBBX26* could interacte with *GhFT*. At the same time, there was no interaction between these genes and *GhSOC1*, *GhAP1* and *GhLFY* (Fig. 7). Therefore, the results suggested that some members of *GhBBXs* regulated the flowering time by interacting with *GhFT*.

### Expression pattern of *GhBBXs* under multiple stress

The analysis of cis-acting elements in the promoter region showed that *GhBBXs* might be related to the abiotic stress, and some studies have shown that *BBX* genes play a positive role in abiotic stress resistance [23, 32]. Therefore, based on the transcriptomic data of TM-1, we further analyzed the expression patterns of *GhBBX* under PEG and NaCl stresses. The results showed that *GhBBXs* clustered mainly into three categories (I-III) based on a hierarchical clustering analysis according to expression features (Fig. 5B). The *GhBBX* genes that belonged to groups II and III responded to PEG and NaCl, especially at 12 h after treatment, showing significantly downregulated expression. 11 *GhBBXs* belonging to group I showed universally low expression after stress treatment.

**Fig. 8:**
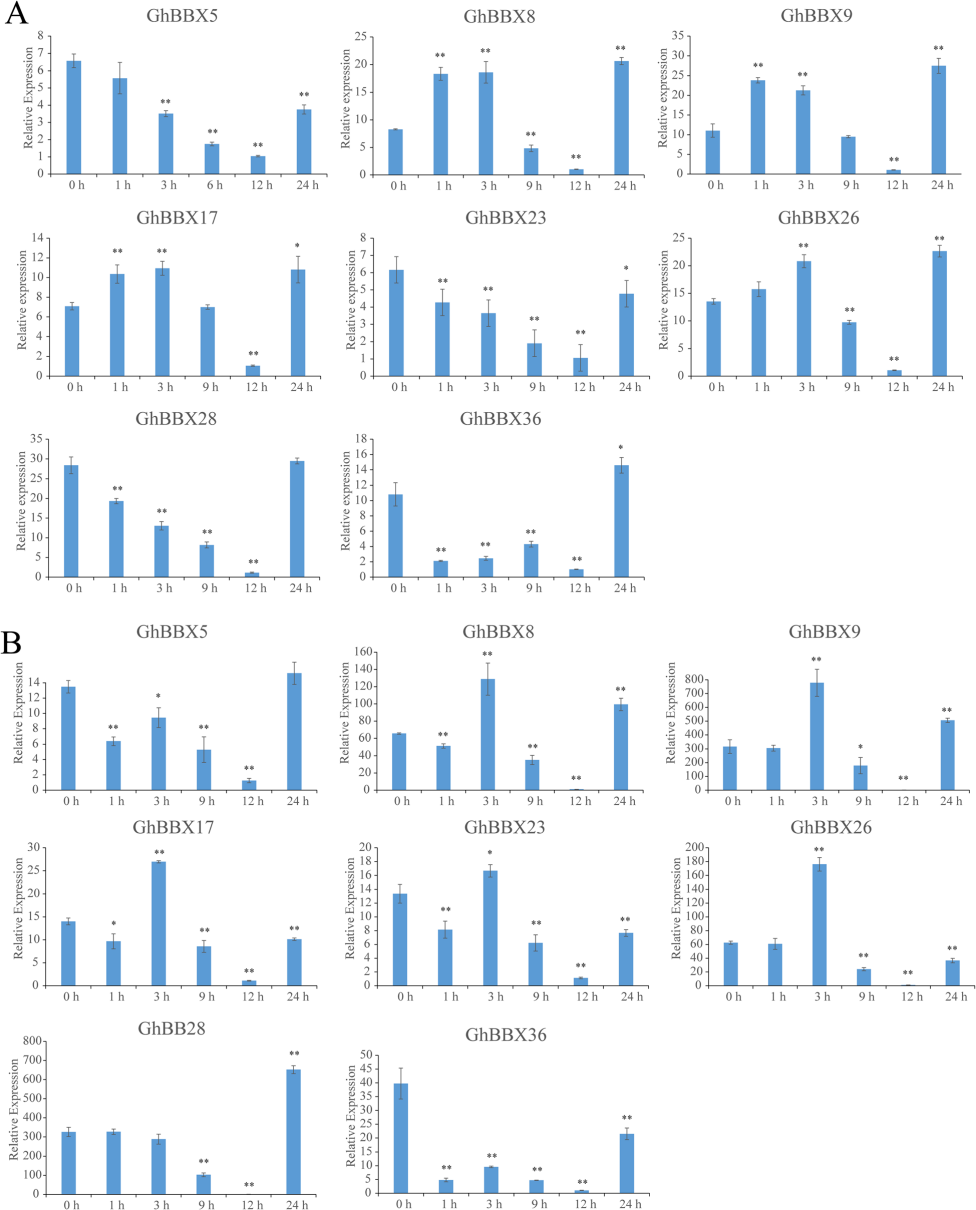
Expression levels of *BBX* genes under PEG and NaCl treatment. (**A**: PEG treatment, **B**: NaCl treatment). The labels 1 h, 3 h, 6 h, 9 h, 12 and 24 h presented hours after PEG and NaCl treatment, with 0 h representing the control sample. The single and double asterisks represent significant differences from the control sample at the 0.05 and 0.01 levels, respectively. The error bars show the standard deviations of three biological replicates

Based on the transcriptomic data, *GhBBX5*, *GhBBX8*, *GhBBX9*, *GhBBX17*, *GhBBX23*, *GhBBX26*, *GhBBX28* and *GhBBX36* which belonged to group II and whose expression levels increased after stress treatment, were selected for qRT-PCR verification. As shown in Fig. 8, PEG stress affected the activity of *GhBBXs*. *GhBBX5*, *GhBBX23* and *GhBBX28* responded to PEG treatment at 1 h, after which their expression was downregulated continuously until a minimum point was reached at 12 h. Afterwards, their expression increased at 24 h. The expression of *GhBBX8*, *GhBBX9*, *GhBBX17* and *GhBBX26* increased at the beginning, after which it decreased but then increased again. The minimum expression level occurred at 12 h. *GhBBX36* was the only gene whose expression abruptly decreased but then increased, followed by a sudden increase after reaching the minimum at 12 h. Under NaCl stress treatment, *GhBBX5*, *GhBBX8*, *GhBBX9*, *GhBBX17*, *GhBBX23*, *GhBBX26* and *GhBBX36* showed the same expression pattern. The expression of these genes quickly decreased but then increased. Afterwards, their expression reached a minimum at 12 h followed by an increase. *GhBBX28* was the only gene whose expression began at 9 h, after which it decreased; after reaching a minimum at 12 h, the expression increased. Taken together, the results showed that *GhBBXs* could respond to plant abiotic stresses, and at the same time, it provided potential candidate genes for further study.

### Expression pattern of *GhBBXs* under phytohormone

**Fig. 9:**
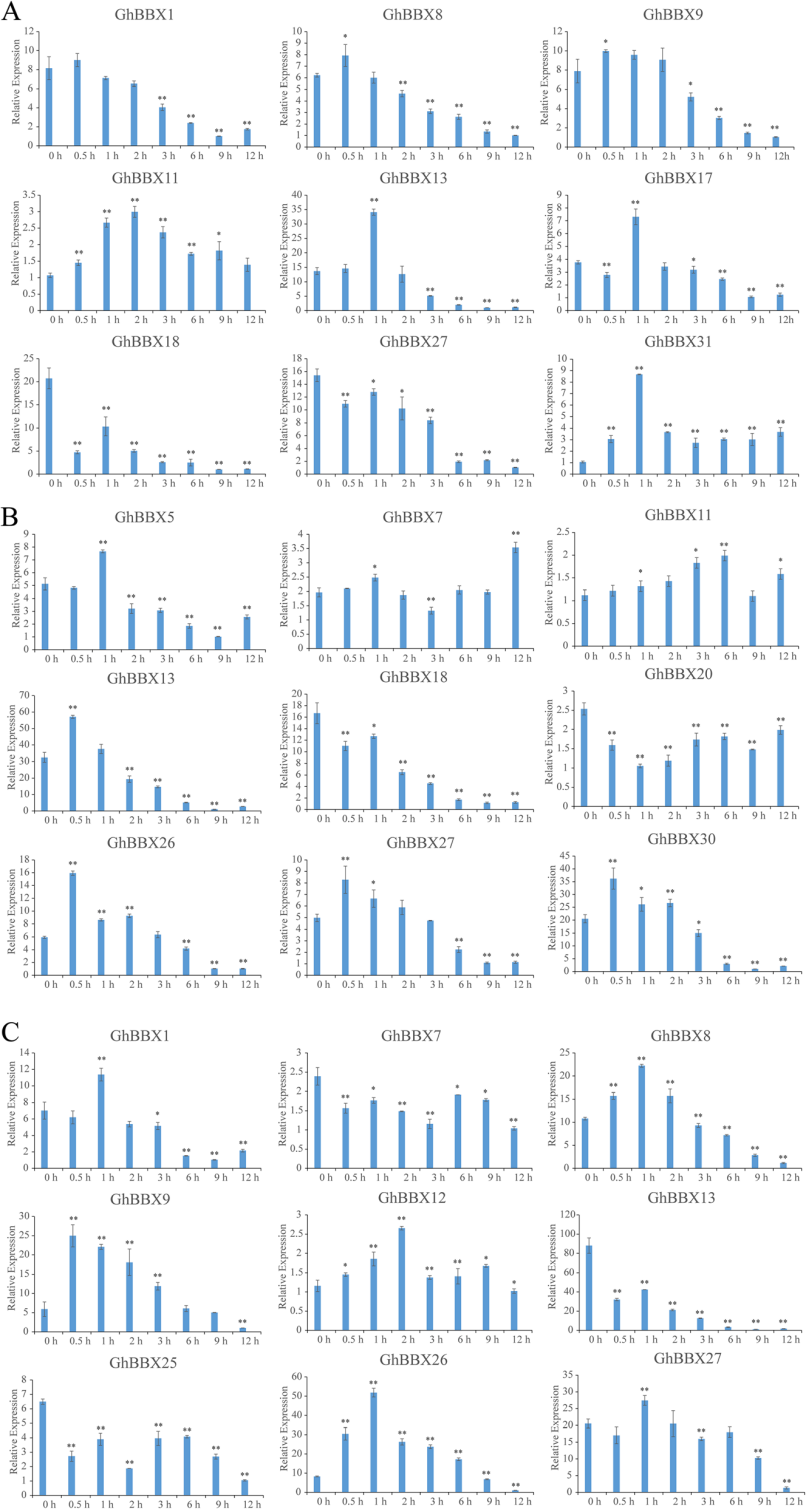
Expression profiles of *BBX* genes in cotton plants treated with exogenous hormones in hydroponic culture. (**A**: 5 µM ABA treatment **B**: 5µM GA treatment **C**: 5µM IAA treatment) The labels 0.5 h, 1 h, 2 h, 3 h, 6 h, and 12 h indicate the time (hour) after treatments. The single and double asterisks represent significant differences from the control sample at the 0.05 and 0.01 levels, respectively. The error bars show the standard deviations of three biological replicates

In order to study the response of *GhBBXs* to phytohormone, 9 genes containing corresponding hormone-response elements in their promoters were selected for qRT-PCR experiment to further analyze the transcript levels of *BBX* genes under three different hormone treatments (Fig. 9). After treated with ABA (Fig. 9 A), the expression of most of these genes increased early (mainly from 0.5 to 2 h), followed by a decrease. The expression of three genes decreased early, after which it increased and finally decreased with time. Under GA treatment (Fig. 9B), 4 expression patterns were found. the expression of most of these genes increased early, peaking at 0.5 or 1 h after GA application, after which their expression decreased over time. The expression of two genes (*GhBBX18* and *GhBBX20*) was downregulated at all time points, and the expression of one gene, *GhBBX11*, was upregulated at all time points except the 9 h time point. The expression of *GhBBX7* was upregulated early on, after which it decreased and then increased. In response to IAA treatment (Fig. 9 C), these genes could be divided into 2 major patterns on the basis of their expression characteristics. The expression of most of these genes increased early, followed by a decrease, peaking from 0.5 to 2 h. Three genes (*GhBBX7*, *GhBBX13* and *GhBBX25*) presented downregulated expression at all time points, reaching a minimum at 12 h.

## Discussion

### Expansion of *BBX* genes in cotton

Polyploidization is the primary mechanism of species formation and plant adaptation to the external environment [33]. The allotetraploid species *G. hirsutum* has undergone many gene duplication events, and the number of genes has increased throughout in the whole evolutionary process, which make it a perfect plant species to study the formation of polyploid in the whole evolutionary process [34, 35]. *G. hirsutum* is an allotetraploid AD genome composed of A genome and D genome. The diploid cotton of A genome is mainly distributed in Asia and Africa, and the cotton of D genome is mainly distributed in America. The AD genome is formed by 1.5 million years of natural hybridization and chromosome doubling of the A genome and the D genome [26]. In this study, in total, 17, 18, 37 and 33 putative *BBX* sequences were identified in *G. arboreum*, *G. raimondii*, *G. hirsutum* and *G. barbadense*, respectively, via genome-wide analysis. The sum of *BBX* genes in *G. arboreum* (17) and *G. raimondii* (19) was about equal to the number of those in *G. hirsutum* (37). Collinearity analysis showed that most homologous gene pairs of the *BBX* gene family were amplified by segmental replication, which meant segmental replication was the main way for the *BBX* gene family to evolve.

### Conservation and evolutionary of *BBX* genes

The conservation of biological function was based on the conservation of structural sequences. We analyzed the conservative motifs and exon/intron structure of *G. hirsutum*, and classified it according to the conservative structure. As shown in Figs. 4 and 9*GhBBX* members were classified into class A, and two *GhBBX* members were classified into class B. All the members in these two categories contained two BBXs plus the CCT domain. The remaining 26 *GhBBX* members carried both B-BOX1 and B-BOX2, which could be divided into C, D and E class according to their gene structure and motif. The remaining 2 domains, B-BOX1 and BOX1 plus CCT, which appeared in *Arabidopsis* did not exist in cotton. To sum up, the gene composition of these *GhBBX* was similar to that of *Arabidopsis* and there were also differences. We speculated that the loss of the other two gene structures in upland cotton might be caused by evolution.

In the process of evolution, it must be under the pressure of natural selection. We calculated the *Ka*/*Ks* ratio between the At and Dt subgenomes of *G. hirsutum* and their corresponding ancestral A and D diploid genomes to evaluate the selection pressure of homologous genes pairs (Table S3). The results showed that the *Ka*/*Ks* values of most *BBX* homologues gene pairs were less than 1. Therefore, we speculated that most of the *BBX* genes had been purified selection throughout the long-term evolutionary, resulting in segmental replication, which might cause the gene to maintain its original function unchanged.

### Cis-acting elements of *BBXs* in *G. hirsutum*

Promoters play an essential role in the regulation of plant gene expression. In this study, we extracted the promoter sequence of 2 kb upstream of the transcriptional initiation site of *GhBBXs* and statistically analyzed the cis-acting elements. As shown in Table S4, we identified a variety of stress-related cis-acting elements in the cotton *BBX* gene promoter, and we speculated that *GhBBX* genes might be related to a variety of external stresses. The result of the qRT-PCR experiment showed that the genes containing these cis-acting elements related to stress enabled plants to respond quickly and regulate the expression of related genes after PEG and salt treatment, so as to avoid further harm to plants. We speculated that they might enhance the resistance to environmental stress by enhancing their resistance to stress in *G. hirsutum*. At the same time, five hormone response elements were identified in cotton *BBX* gene promoter, and some genes containing cis-acting elements of ABA, GA and IAA were tested by qRT-PCR experiment. The results showed that the expression of genes containing hormone response elements changed in different degrees after dredging the corresponding hormone treatment. The promoters of some *BBX* genes contained more than one hormone-responsive cis-acting element. We speculated that these genes might participate in hormone signal transduction and act as transcriptional regulators. Their expression might be regulated by a variety of plant hormones.

### Potential roles of *GhBBX* genes in *G. hirsutum*

An increasing number of studies have shown that *BBX* genes are involved in many aspects such as plant growth and development, as well as in resisting abiotic stress. TM-1 expression profiles were used to evaluate the expression patterns of 37 *GhBBX* genes in different cotton tissues and after external stress treatment. They were classified according to their expression abundance in the transcriptome, which might reflect genes in the same group might have similar functions. The results will provide strong data support for the study of specific genes.

*BBX* genes are involved in the flowering pathway. In *Arabidopsis*, *BBX6*/*COL5* promoted flowering by increasing the expression of *FT* under short-day conditions [19], while *BBX7*/*COL9* negatively regulates flowering by inhibiting the expression of *CO* and *FT* [17]. *BBX32*/*EIP6* might inhibit flowering probably in a way independent of *CO* [36]. According to our findings, *GhBBX8* and *GhBBX26* were the closest homologues of *AtBBX6* in cotton and the expression level in flower organs was significantly higher than that in other organs. Moreover, these genes clustered together with the other four genes, *GhBBX5*, *GhBBX9*, *GhBBX23* and *GhBBX28*, in a heat map of tissue expression specificity. Therefore, we speculated that these genes might be related to flowering. Flowering is closely related to flower bud differentiation and early maturity of cotton. The qRT-PCR experiment results showed, these genes presented significant differences in the leaf and shoot apex of the early-maturing material CCRI50 and the late-maturing material GX11 from one-leaf stage to five-leaf stage at most periods. We validated the interaction of some members of *GhBBXs* with key factors in the cotton flowering pathway by yeast two-hybrid. The results showed that *GhBBX5*, *GhBBX8*, *GhBBX23* and *GhBBX26* could interact with *GhFT*. Preview studies have showed that overexpression of *GhFT* in *Arabidopsis* could lead to early flowering of transgenic plants [28]. Taken together, we speculated that *GhBBX* gene could regulate the flowering time by interacting with *GhFT*.

*BBX* gene is also involved in stress signal transduction. In *Arabidopsis*, *BBX24* was involved in signal transduction in response to salt stress. Compared with wild-type *Arabidopsis* plants, *Arabidopsis* plants overexpressing *BBX24* were more salt-tolerant [20]. The results of qRT-PCR experiment showed that most *BBX* family genes began to be expressed at 1 h after abiotic stress. This rapid response mechanism could protect plants and reduce the damage caused by abiotic stress. Moreover, the *BBX* genes had a common feature: regardless of the changes in their expression at previous stages, the minimum expression of each occurred at 12 h. Taken together, these results indicated the *GhBBX* proteins played an important role in plant resistance to abiotic stress. However, the specific functions and regulatory mechanisms need to be clarified by further investigation.

## Conclusions

In this study, members of the *BBX* gene family were identified in *Gossypium arboreum*, *G. raimondii*, *G. hirsutum* and *G. barbadense*, respectively. The main purpose of our study was to explore the phylogenetic relationships, collinearity, expansion patterns, gene structures, cis-acting elements and expression patterns of the *BBX* family. The transcription of *GhBBXs* in different tissues indicated that some members of *GhBBXs* might play a role in flower bud differentiation of cotton. The qRT-PCR results showed that there were significant differences in *GhBBX* genes in leaves and shoot apex of early-maturing materials and late-maturing materials at most periods. Through the yeast verification of the four key genes in the flowering pathway, *GhBBX* and *GhFT*, *GhSOC1*, *GhLFY*, *GhAP1*, we found that *GhBBX* might interact with *GhFT* to regulate the flowering time. Transcriptome and qRT-PCR analysis showed that *GhBBX* genes were involved in the complex regulatory network of the abiotic stress response. After hormone treatment, it was found that *GhBBX* gene might play an essential role in regulating plant growth and development by regulating a variety of hormone signal pathways. In general, genome-wide analysis of *BBXs* has laid a solid foundation for the functional analysis of *BBX* genes in cotton.

## Materials and methods

### Identification of the *BBX* gene family

The genomes sequences of *G. arboretum* (CRI, version 1.0), *G. barbadense* (HAU, version 1), *G. hirsutum* (HAU, version 1) and *G. raimondii* (JGI, version 2.1) were obtained from the CottonFGD (http://www.cottonfgd.org/) [37]. The BBX protein sequences of *Arabidopsis thaliana* were obtained from the TAIR website (https://www.arabidopsis.org/) [38]. The hidden Markov model (HMM) configuration file of the *BBX* conservative domain (PF00643) was downloaded from the Pfam database (https://pfam.xfam.org/) [39]. HMMsearch program was used to search the *BBXs* in cotton by using the HMM file. The threshold of E-value was 1.0 E^− 10^ [40]. The SMART database (http://smart.embl-heidelberg.de/) was subsequently used to identify whether the candidate protein sequences contain the special domain of *BBX* family [41]. The ExPASy website (http://web.expasy.org/protparam/) was used to predict the amino acid (aa) sequence, molecular weight (MW) and isoelectric point (pI) of the identified BBX proteins[42], and the subcellular localization was predicted through the ProtComp version 9.0 server (http://linux1.softberry.com/berry.phtml).

### Phylogenetic analysis and sequence alignment

Multiple sequence alignment of BBX proteins from the four *Gossypium* species and *Arabidopsis* was performed by ClustalW. By using the MEGA 7.0 program and neighbour-joining (NJ) method, a rootless phylogenetic tree was constructed. Bootstrap replications (1000) were used to evaluate the reliability of the interior branches [43].

### Chromosome location and gene duplication analysis

The physical chromosome locations of all *BBX* genes were obtained from the genome sequence databases of the four *Gossypium* species, and MapChart software was used for visualization [44]. The genome replication gene pairs of *G. hirsutum*, *G. raimondii* and *G. arboreum* were detected by MCScanX software [45]. Circos was used to map the segmental duplication events on chromosomes [46]. TBtools was used to calculate the non-synonymous mutation rate (*Ka*) and synonymous mutation rate (*Ks*) of *BBX* genes replication [47].

### Conserved sequence and gene structure analysis

The exon-intron structure was analyzed on the GSDS (http://gsds.cbi.pku.edu.cn/) based on inputting GFF gene annotation files [48]. The motifs of BBX protein sequences were confirmed by the MEME (http://meme-suite.org/tools/meme), using the following parameters: up to six motifs, the best width of 6 to 50 [48].

### Promoter region cis-acting elements analysis

The promoter sequence 2000 bp upstream of each *GhBBX* gene coding region was retrieved from CottonFGD (http://www.cottonfgd.org/). The predicted cis-acting elements were searched by using the PlantCARE online program (http://bioinformatics.psb.ugent.be/webtools/plantcare/html/) [49].

### Gene expression pattern analysis

The expression levels of *GhBBXs* in different tissues and after abiotic stress responses were obtained from previously reported transcriptome data of *G. hirsutum* TM-1, which were obtained from the NCBI Sequence Read Archive (SRA:PRJNA490626) (https://www.ncbi.nlm.nih.gov/bioproject/PRJNA490626) [50]. TBtools was used to draw a heat map to show the expression patterns of *GhBBX* genes [47].

### Plant materials and treatments

The early-maturing cotton cultivar CCRI50 and the late-maturing cotton cultivar Guoxinmian11(GX11) were grown in the field in Anyang, Henan Province, China. Leaf and shoot apex samples were taken at one, two, three, four and five-leaf stages. The samples were collected and frozen immediately in liquid nitrogen, after which they were stored at − 80 °C.

The *G. hirsutum* cultivar Texas Marker-1 (TM-1) was used for stress and hormone treatments of seedlings in a climate-controlled greenhouse (light/dark cycle: 16 h at 28 °C/8 h at 22 °C). The seedlings were grown in hydroponic solution (pH 6.2) at the four-week-old of the three-leaf stage were treated with consisting of sodium chloride (300 mmol/L), polyethylene glycol (PEG) 6000 (30 %), 5 µM abscisic acid (ABA), 5 µM gibberellic acid (GA) or 5 µM auxin (IAA), respectively. Control plants were only treated with hydroponic solution. Leaves were harvested at 0 h, 1 h, 3 h, 9 h, 12 h, 24 h after stress treatments, and leaves were harvested at 0 h, 0.5 h, 1 h, 2 h, 3 h, 6 h, 9 h, and 12 h after hormone treatments. The samples were collected and frozen immediately in liquid nitrogen, after which they were stored at − 80 °C.

### RNA extraction and qRT-PCR analysis

Total RNA was extracted from the collected samples using the RNA-prep Pure Plant Kit (TIANGEN, Beijing, China). Reverse transcription experiment was carried out using a PrimeScript RT Reagent Kit (Takara, Dalian, China). The gene-specific primers for qRT-PCR (Table S1) were designed with Oligo 7.0 software; *G. hirsutum Actin* (*GhActin*) was used as an internal control. qRT-PCR (Promega, Madison, WI, USA) (three biological replicates) was performed on an ABI 7500 real-time PCR system (Applied Biosystems, USA). The scheme was carried out as follows: step 1, 95 °C for 30 s; step 2, 40 cycles of 95 °C for 5 s followed by 60 °C for 34 s; and step 3, melting curve analysis. The relative expression levels of *GhBBXs* were calculated by 2^−ΔΔCT^ method, and statistical analyzed by T-tests.

### Yeast two-hybrid assay

The full-length sequences of *GhBBX5*, *GhBBX8*, *GhBBX9*, *GhBBX23*, *GhBBX26* and *GhBBX28* were cloned into pGADT7 vector, and the coding sequences of *GhFT*, *GhSOC1*, *GhAP1* and *GhLFY* were cloned into pGBKT7 vector with the gene-specific primers (Table S5). Different combinations of recombinant plasmids pGADT7 and pGBKT7 were co-transformed into yeast strain Y2HGold and cultured on DDO (SD/-Leu/-Trp) plate for 3 days. Three independent colonies on the DDO plate were selected and the interaction was further detected on the QDO (SD/-Leu/-Trp/-His/-Ade) plate.

## Supplementary information

Additional file 1: **Table S1.** Primer pairs used in the qRT-PCR analysis.

Additional file 2: **Table S2. **Detailed physicochemical characteristics and Gene location of BBX proteins of *A. thaliana*, *G. arboreum*, *G. raimondii*, *G. hirsutum* and *G. barbadense*. The pI and MW were computed by ExPASy website. Subcellular localizations of BBX proteins were predicted by protcomp version 9.0 server. Gene locus information for *BBXs* in *G. arboreum*, *G. raimondii*, *G. hirsutum* and *G. barbadense* was obtained from gene annotation.

Additional file 3: **Table S3. ***Ka*/*Ks* ratios and duplicate types of gene pairs between the A and D subgenomes of allotetraploid cottons and their corresponding ancestral A and D diploid genomes, respectively. The homologous gene pairs were identified by the results of BLAST and collinearity analysis. When both *Ka* and *Ks* were equal to 0, *Ka*/*Ks* was considered equal to 1. When only *Ks* was equal to 0, it was marked as *Ka* > > *Ks*. Segmental means that the gene might arise from segmental duplication.

Additional file 4: **Table S4. **Statistical results of phytohormone and stress response cis-acting elements in the promoter segments of *BBXs*. These cis-acting elements were identified using PlantCARE software with the upstream 2000-bp sequences of cotton *BBXs.*

Additional file 5: **Table S5.** Primer pairs used for gene clone in yeast two-hybrid assay

## Data Availability

The data included in this article and the additional files are available. The transcriptome datasets of G. hirsutum TM-1 are under the accession number in PRJNA490626 NCBI.
